# Evaluating the Intensity of Muscle Contraction by Near‐Infrared Spectroscopy, a Potential Application for Scaling Muscle Spasm

**DOI:** 10.1002/jbio.70020

**Published:** 2025-04-03

**Authors:** Mehdi Nourizadeh, Yekta Saremi, Amir Parham Pirhadi Rad, Sepideh Mortezanezhad, Iman Amani Tehrani, Jocelyn Bégin, Maria Juricic, Kishore Mulpuri, Babak Shadgan

**Affiliations:** ^1^ International Collaboration on Repair Discoveries Vancouver Canada; ^2^ Department of Orthopaedics University of British Columbia Vancouver Canada; ^3^ Department of Pathology & Laboratory Medicine University of British Columbia Vancouver Canada; ^4^ School of Biomedical Engineering University of British Columbia Vancouver Canada; ^5^ Department of Physical Therapy University of British Columbia Vancouver Canada; ^6^ BC Children's Hospital, Department of Orthopaedic Surgery University of British Columbia Vancouver Canada

**Keywords:** electromyography, muscle contraction, near infrared spectroscopy, spasticity, tissue oxygenation

## Abstract

Muscle spasticity, common in conditions such as cerebral palsy, spinal cord injury, and multiple sclerosis, is traditionally assessed using the Modified Ashworth Scale, which lacks consistency. This study evaluates near‐infrared spectroscopy (NIRS) as a non‐invasive tool for measuring muscle contraction intensity. Thirty‐seven healthy adults performed isometric contractions at varying intensities (15%, 30%, 45%, and 60% of maximal voluntary contraction), with NIRS sensors monitoring changes in the Tissue Oxygenation Index (TOI) and electromyography (EMG) measuring muscle activity. Results demonstrated a significant negative correlation between contraction intensity and ΔTOI, indicating that higher contraction levels resulted in greater reductions in muscle oxygenation. Additionally, a multinomial logistic regression model confirmed that TOI could reliably predict contraction intensity (*p* < 0.001). This technique could provide real‐time, objective data for spasticity assessment, potentially improving treatment plans.

## Graphical abstract

Near‐infrared spectroscopy (NIRS) offers a novel, non‐invasive method to assess muscle spasticity. By measuring the Tissue Oxygenation Index (TOI) during different levels of muscle contraction, this study explores the feasibility of NIRS as an objective tool for spasticity evaluation. The findings contribute to advancing quantitative assessments of muscle function, potentially improving clinical diagnosis and treatment strategies.

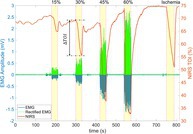

**Abbreviations**
CP: Cerebral Palsy
EMG: Electromyography
HHb: Deoxygenated Hemoglobin
MVC: Maximal Voluntary Contraction
NIRS: Near Infra‐red Spectroscopy
O_2_Hb: Oxygenated Hemoglobin
TOI: Tissue Oxygenation Index

## 1 Introduction

Muscle spasticity is a prevalent symptom in a variety of neurological and neuromuscular disorders, including spinal cord injury, stroke, cerebral palsy (CP), and multiple sclerosis. This condition is characterized by involuntary and excessive muscle contractions, leading to complications such as pain, reduced functioality, limb deformities, and an impaired ability to perform daily activities [1].

In pediatric populations, muscle spasticity is a significant concern, as it can lead to substantial functional impairments and developmental challenges. For instance, muscle spasticity is a primary concern in CP, affecting approximately 80% of patients [2]. Studies indicate that spasticity in the CP population may progress during the early years, often peaking around the age of four [3], highlighting the importance of early detection and management to mitigate side effects and prevent secondary complications. Considering that the severity of spasticity in CP patients ranges from mild to uncontrollable spasms, treatments also vary widely; therefore, selecting an appropriate treatment plan and evaluating the effectiveness of the chosen therapeutic intervention hinge on an objective assessment of the patient's muscle spasticity [1, 4]. Yet, despite its prevalence, achieving a non‐invasive, accurate, and objective measurement of muscle spasticity remains an ongoing challenge [4, 5, 6].

Currently, there are quantitative and qualitative methods for measuring spasticity that are commonly used in clinical settings, including the Modified Ashworth Scale, the Pendulum test, and Isokinetic dynamometers [4, 7]. However, current methods have limitations in terms of accuracy and objectivity, are difficult to perform, and rely heavily on the expertise and experience of the medical practitioner, casting doubt on the reliability of these tests [8, 9, 10]. To date, reliable techniques for the objective measurement of muscle spasticity at the bedside are lacking [5].

This study investigated the potential application of near‐infrared spectroscopy (NIRS) to quantify the intensity of muscle contractions as a simulation of muscle spasms in a cohort of healthy adult individuals. NIRS is a non‐invasive optical technology that uses near‐infrared light to study tissue oxygenation and hemodynamics by monitoring changes in the concentrations of oxygenated and deoxygenated hemoglobin. When used in a spatially resolved configuration, NIRS can measure real‐time tissue oxygen saturation index (TOI) percentage. The science of NIRS hinges on fundamental principles of optics and photonics, where near‐infrared light penetrates biological tissues and is absorbed by tissue chromophores, primarily oxygenated (O_2_Hb) and deoxygenated hemoglobin (HHb). Variations in photon absorption at specific wavelengths are detected and processed using software‐based mathematical algorithms, employing a modified Beer–Lambert law to generate real‐time concentration changes for each chromophore. Using this data, real‐time changes in tissue oxygenation and hemodynamics can be derived. Several studies have demonstrated that transcutaneous NIRS can effectively detect changes in skeletal muscle oxygenation and total blood volume following muscle contraction [11, 12, 13, 14, 15].

A twofold hypothesis guided our study. First, we hypothesized there is a significant correlation between muscle oxygenation levels, as measured by NIRS, and the intensity of muscle contractions in healthy adults—a relationship previously observed in our study and that of others. Second, we hypothesized that the NIRS‐derived muscle TOI could reliably predict muscle contraction intensity. If this relationship is confirmed, we propose using transcutaneous NIRS to scale muscle spasms. Developing a non‐invasive method for the objective assessment of muscle spasticity in patients with neuromuscular conditions, including children with CP, will enhance the accuracy of diagnosis and the ability to monitor treatment effectiveness.

## 2 Methods

### 2.1 Study Design

This study utilized a controlled experimental design to investigate the correlation between the forearm muscle TOI and muscle contraction intensities.

### 2.2 Participants

The study received institutional ethics approval, and informed written consent was obtained from all volunteers before participating. All procedures complied with the Declaration of Helsinki. Healthy adult volunteers from the local community were recruited. Individuals with a history of neuromuscular disorders, upper limb injuries, or contraindications to NIRS and electromyography (EMG) were excluded.

### 2.3 Instruments

**FIGURE 1:**
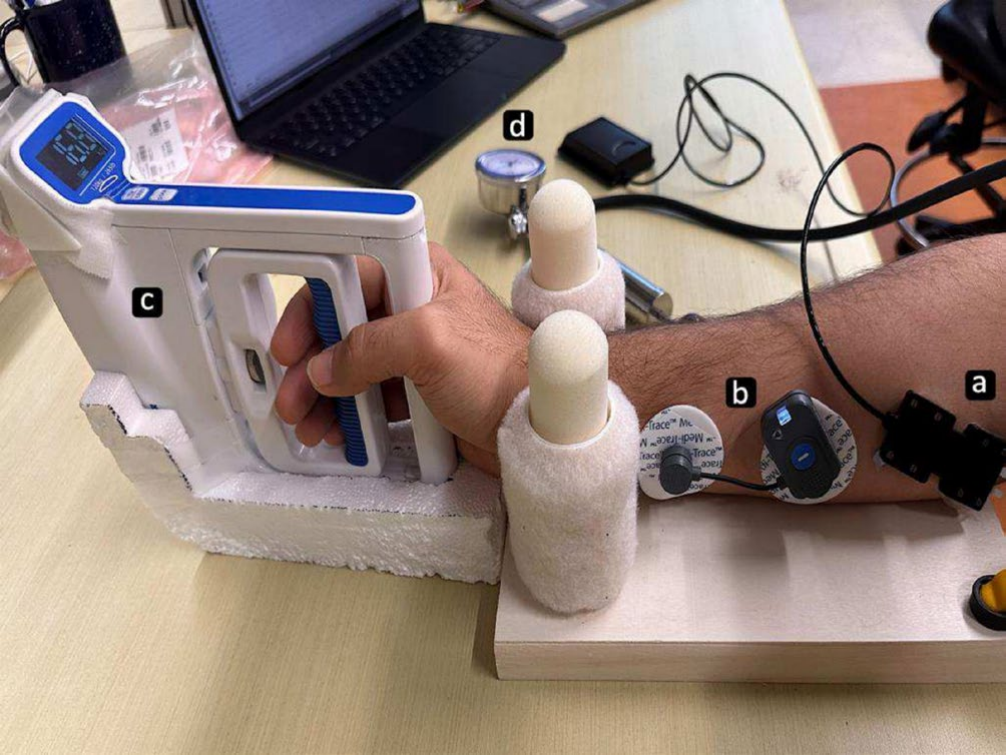
The experimental setup. (a) NIRS sensor placed and fixed on the flexor carpi radialis muscle, (b) surface EMG sensor, (c) digital dynamometer, and (d) pneumatic tourniquet device.

We utilized a transcutaneous NIRS device (Portalite, Artinis Medical Systems, BV, Netherlands) and a wireless surface EMG sensor (BTS‐EMG Analyzer v. 2.10.43.0) to record the TOI and electrical activations of the flexor carpi radialis muscle of their dominant hand during different degrees of isometric muscle contractions induced and measured by a digital dynamometer (Handexer, HFEH20WBAB, US). The experimental setup has been illustrated in Figure 1.

### 2.4 Experimental Protocol

First, the maximal voluntary contraction (MVC) of the flexor muscles was measured using the dynamometer. Four contraction intensities (15%, 30%, 45%, and 60% MVC) were then calculated based on the MVC. The experimental protocol commenced with a 3‐min baseline measurement followed by sequential isometric contractions at the predetermined intensities. Each contraction was maintained for 30 s and interspersed with a 90‐s rest period. This resting period is required to allow muscle fibers to be adequately oxygenated and recover before the next contraction phase begins.

After the last contraction, participants rested for three additional minutes. The session ended with a 30‐second forearm muscle ischemia induced by a pneumatic tourniquet placed around the arm. The cuff pressure was raised above 20 mmHg over the systolic blood pressure for each participant to induce complete ischemia. After the tourniquet was deflated, the participant could rest for 30 s.

### 2.5 Outcome Measures

**FIGURE 2:**
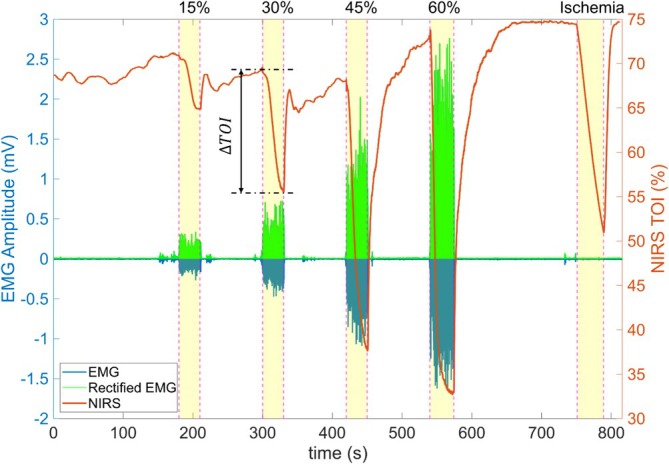
A representative figure showing the relationship between NIRS and EMG signals at different muscle contraction intensities.

The primary outcome measure in this study was the level of spasticity, categorized into four contraction intensity levels: 15%, 30%, 45%, and 60% of maximal voluntary contraction (MVC). These levels were assessed using NIRS to monitor changes in the muscle TOI. The decline in TOI from the beginning to the end of each contraction phase was ∆TOI, which was recorded as an indicator of muscle oxygenation. The study aimed to evaluate the relationship between ∆TOI and spasticity levels, providing a predictive measure for spasticity assessment (Figure 2).

### 2.6 Data Analysis

The data were analyzed to assess the relationship between muscle contraction intensity and changes in muscle TOI using NIRS.

A multinomial logistic regression model was conducted to investigate whether ∆TOI can predict muscle contraction levels. The dependent variable, spasticity level, had four ordered levels (15%, 30%, 45%, and 60%), and the predictors included ∆TOI (numeric), sex (male vs. female), and age (numeric). The logistic link function was used to model the cumulative probabilities of being in higher contraction levels.

Model validation was performed using the Akaike Information Criterion (AIC) and Residual Deviance, and the significance of each predictor coefficient was tested using the t‐test. A significance threshold of *p* < 0.05 was applied. Muscle activity data, recorded in millivolts, were extracted using BTS‐EMG Analyzer (v. 2.10.43.0) at a sampling rate of 1000 Hz. A MATLAB‐based signal processing algorithm was used for outlier handling, EMG envelope detection through filtration and optimization, and extraction of temporal signal features. All statistical analyses were conducted using R‐Studio.

## 3 Results

### 3.1 Muscle Oxygenation Changes During Muscle Contractions

**FIGURE 3:**
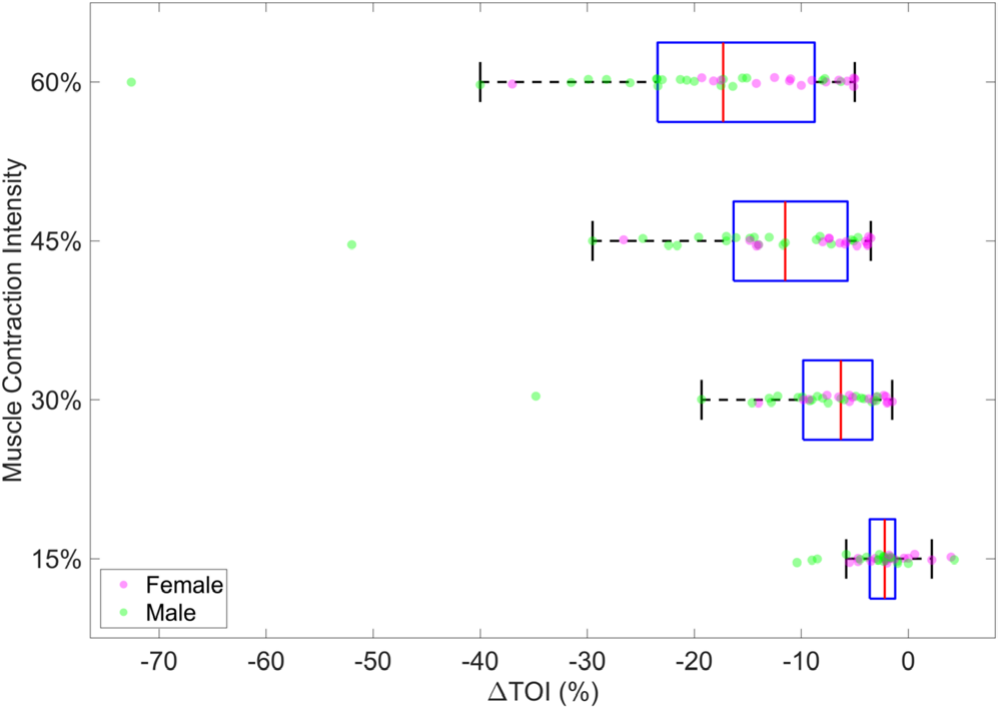
The box plot illustrates the distribution of tissue ∆TOI percentages across different contraction intensities.

Thirty‐seven healthy adult participants were recruited, including 57% male and 43% female. The bivariate analysis and computation of the mean ΔTOI in each contraction category in the entire sample revealed a negative association between ΔTOI and the intensity of contraction (Figure 3). The mean ΔTOI were −2.45 for 15% contraction, −7.76 for 30%, −12.77 for 45%, −18.44 for 60% contraction, and −10.61% at Ischemia.

### 3.2 Confirmation of the Different Levels of Contractions Using Electromyography and Its Relationship With the Tissue Oxygenation Index

**FIGURE 4:**
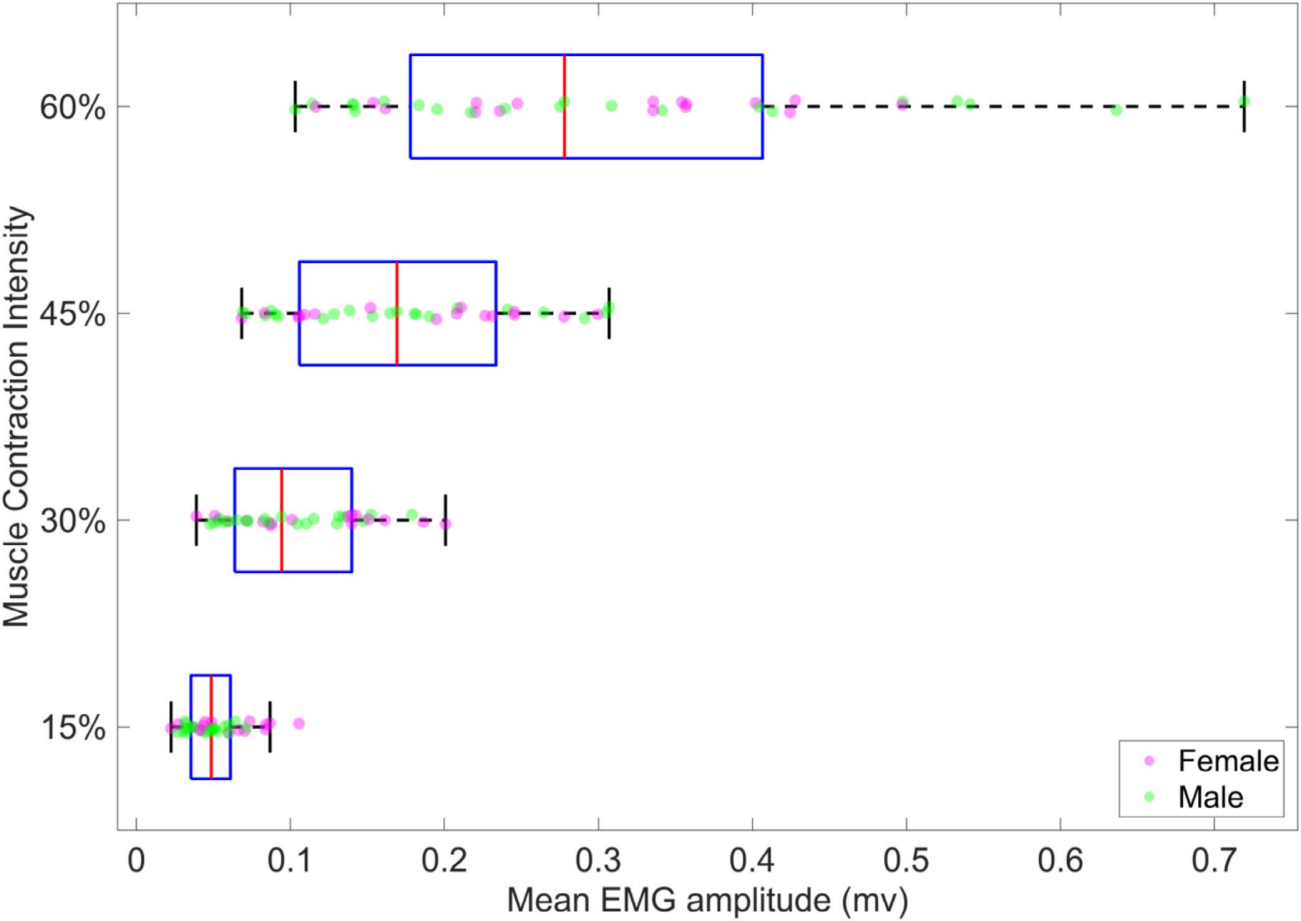
Depicting EMG amplitude at different contraction intensities, a confirmation of the existence of different contraction levels.

The mean EMG amplitude values were 0.0509 for 15% contraction, 0.10 for 30% contraction, 0.173 for 45% contraction, 0.308 for 60% contraction, and 0.004 for Ischemia. Figure 4 demonstrates the amplitude of EMG at various voluntary contractions.

**FIGURE 5:**
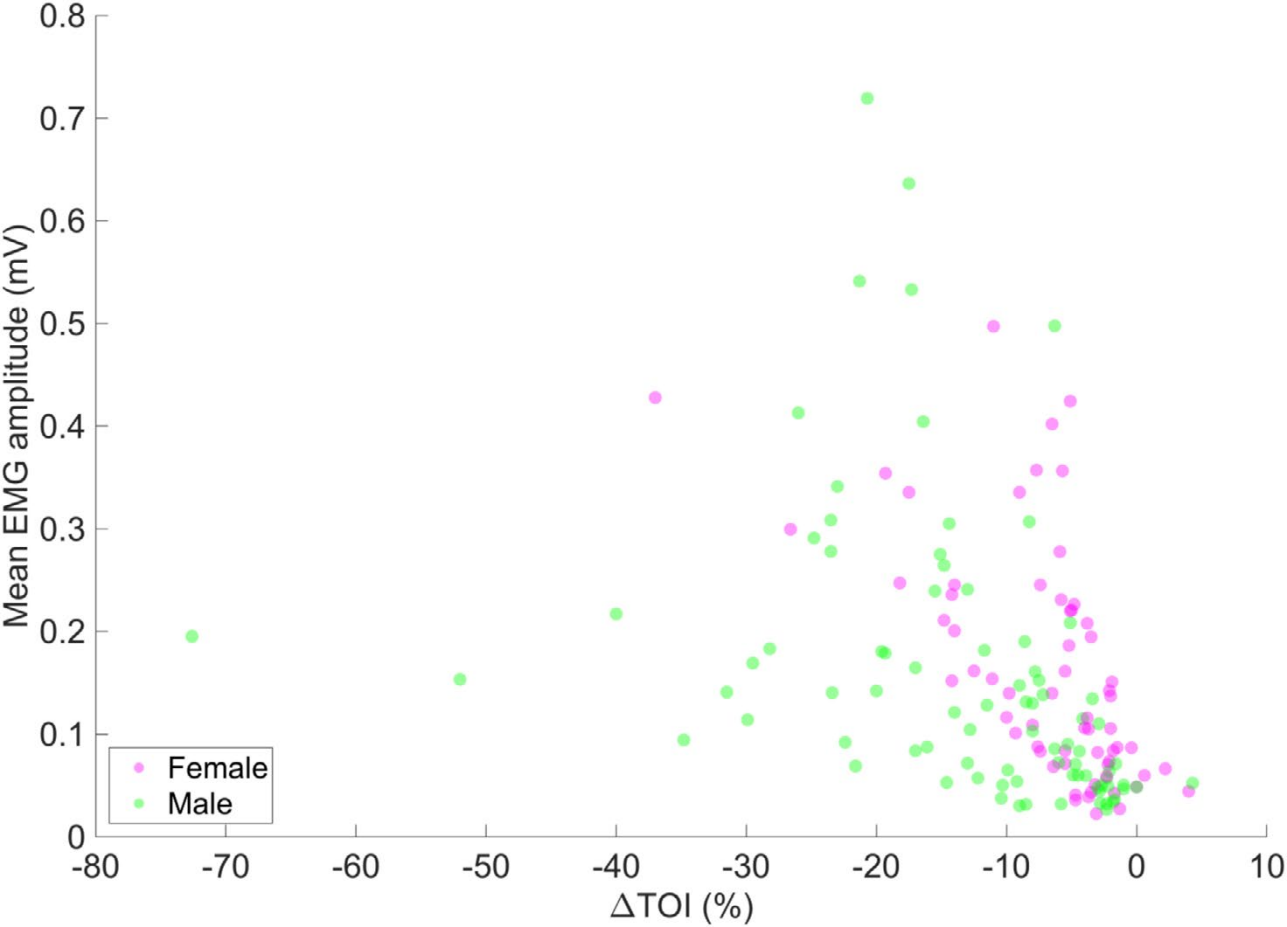
A scatterplot illustrating the relationship between ∆TOI and EMG amplitude.

Furthermore, we explored the relationship between ∆TOI and EMG amplitude and calculated the correlation between them. The correlation coefficient of −0.495 indicates a moderate negative linear relationship between ΔTOI and EMG amplitude (Figure 5).

### 3.3 Estimating Contraction Levels Using NIRS

**FIGURE 6:**
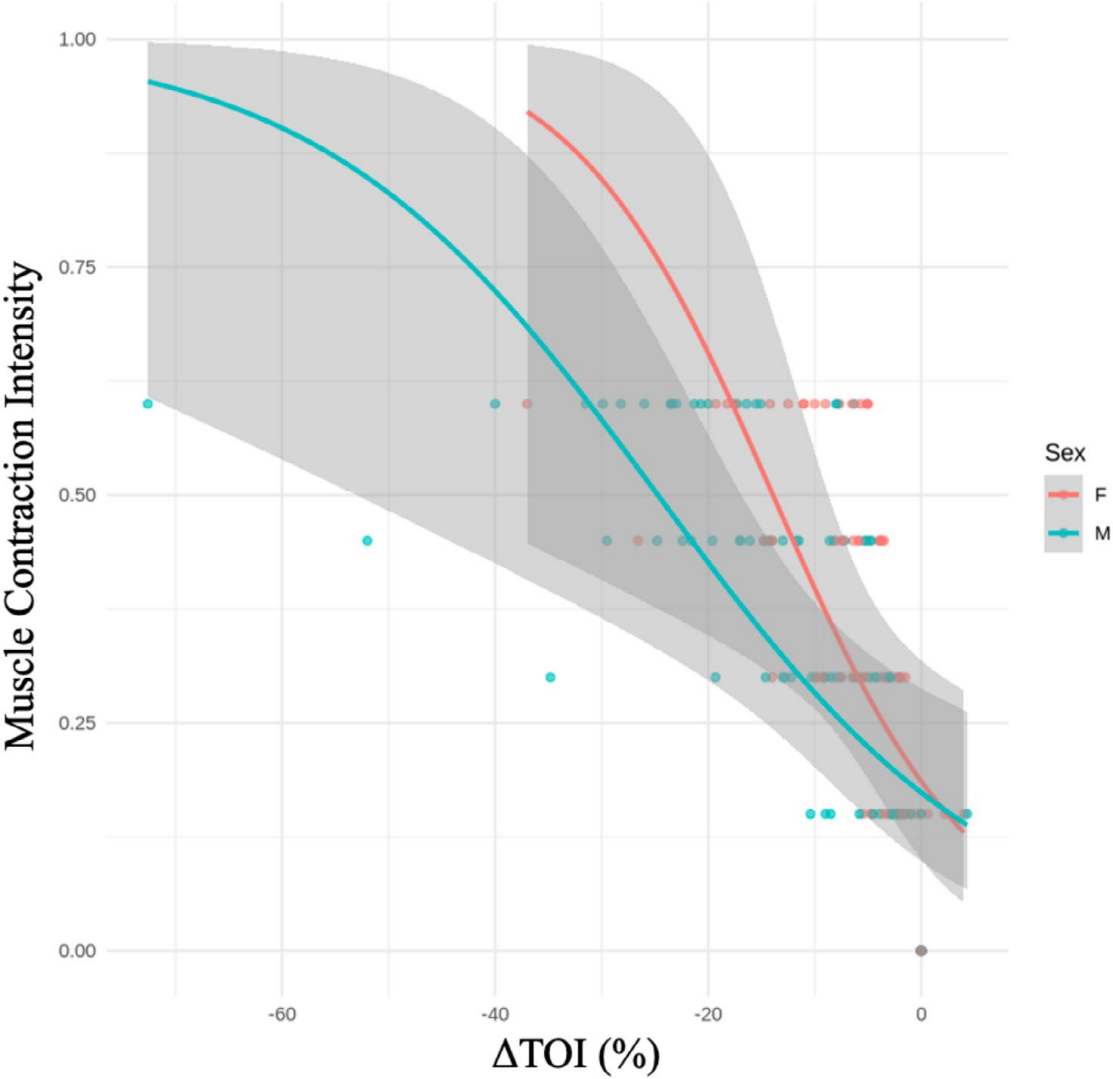
Logistic Regression Model; the relationship between ΔTOI values and contraction intensity levels, with separate curves for males (blue line) and females (red line). Each dot represents a data set, and the grey area illustrates the Confidence Interval.

Multinomial logistic regression analysis identified ∆TOI as a significant predictor of spasticity levels (*p* < 0.001). The model exhibited a good fit, with a residual deviance of 456.75 and an AIC of 470.75. A decrease in ∆TOI was associated with higher spasticity levels, with each 1‐unit decrease increasing the log odds of being in a higher spasticity category by 0.259, after controlling for sex and age. Additionally, a significant inverse relationship was observed between contraction intensity and TOI, indicating that greater muscle contraction resulted in a more pronounced reduction in tissue oxygenation. These findings support ∆TOI as a reliable biomarker for quantifying spasticity intensity (Figure 6). The model's coefficients, standard errors, and *t*‐values are summarized in Table 1.

**TABLE 1:** The summary of Logistic Regression Model's coefficient, standard errors and *t*‐values.

Predictor	Coefficient (value)	SE	*t*‐value	Significance
NIRS ‐B	−0.259	0.028	−9.13	*p* < 0.001***
Sex (M)	−1.077	0.306	−3.52	*p* < 0.01**
Age	0.009	0.014	0.67	Not significant (*p* > 0.05)

## 4 Discussion

This study explores the application of NIRS as a non‐invasive tool for quantifying muscle contraction intensity, with a particular focus on its potential utility in evaluating muscle spasticity. Our findings confirm that NIRS can reliably detect changes in muscle oxygenation during voluntary contractions, demonstrating a systematic decline in the muscle TOI with increasing contraction intensity. However, a critical question remains: how does this relationship contribute to the objective assessment of muscle spasticity?

### 4.1 Utility of NIRS for Muscle Spasm Quantification

Our results showed a systematic decrease in ΔTOI during voluntary muscle contraction, and the magnitude of ΔTOI became smaller as the percentage of voluntary contraction increased. Lower ΔTOI values were linked to higher contraction intensities, suggesting a potential relationship between the intensity of muscle contraction and the level of muscle tissue oxygenation percentage [16].

Although previously demonstrated by Paiziev et al. and McNeil et al. [17, 18], our study uniquely suggests utilizing this relationship to quantify muscle contraction as a simulation of muscle spasms in a clinical setting. Unlike voluntary contractions, muscle spasms are involuntary and often unpredictable, making objective measurement challenging. Our results indicate that NIRS may detect fluctuations in muscle oxygenation that are not solely driven by voluntary movement, suggesting its potential utility in assessing involuntary muscle activity [19]. However, further investigation is required to validate its application in spasticity‐related conditions.

The observed TOI reductions provide valuable insights into the metabolic demands of muscle contractions. At 15% MVC, TOI decreased by an average of −2.45%, whereas at 60% MVC, the reduction reached −18.44%, highlighting a graded response to increasing contraction intensity. This gradient underscores the capability of NIRS to differentiate between mild and intense contractions, a property that could be utilized for assessing spasticity severity. In clinical settings, spastic contractions are often unpredictable and non‐uniform; therefore, real‐time NIRS‐based TOI monitoring could facilitate the detection and characterization of transient and sustained muscle contractions, offering an objective measure of spasticity [20].

Previous studies have shown that conventional spasticity assessment tools, such as the Modified Ashworth Scale (MAS), often lack the sensitivity to detect subtle changes in muscle function [9]. By integrating TOI measurements, clinicians can obtain a more comprehensive assessment of spastic muscle behavior, particularly in cases where visual or manual evaluations are insufficient. This approach could enhance diagnostic precision and improve treatment monitoring in patients with spasticity.

While our experimental model used controlled contractions to simulate muscle spasticity, the clinical application of NIRS differs slightly. In practice, clinicians would not use spasticity levels to predict TOI values; rather, TOI would be used to quantify the magnitude of spasticity. The reduction in TOI reflects increased muscle activation and oxygen demand, making it a useful biomarker for assessing spasticity severity. Therefore, while our multinomial logistic regression model demonstrates the strong relationship between TOI and contraction intensity, its clinical value lies in its ability to provide real‐time, objective measurements of spasticity severity in neuromuscular patients.

### 4.2 TOI as an Index for Spasticity Assessment

Building on our data, we demonstrate that TOI can serve as a predictive index for assessing muscle spasticity levels. The logistic regression model used in our study shows that TOI values can reliably differentiate between various contraction intensities, supporting its application as a quantitative measure of muscle spasticity. The significant inverse relationship between contraction intensity and TOI (*p* < 0.001) suggests that lower TOI values correspond to higher spasticity levels.

As shown in Figure 6, sex differences in spasticity levels are evident, with the red curve (females) positioned slightly above the blue curve (males). This indicates that females tend to exhibit higher spasticity levels for the same TOI value, with the difference being more pronounced at lower ∆TOI values [10]. However, the wider confidence intervals in this range suggest greater variability and increased uncertainty in predictions. Furthermore, the overlapping confidence intervals indicate that sex‐based differences may not be statistically significant, while a trend is observed.

The distribution of individual data points further supports this trend, with higher ∆TOI values clustering at lower spasticity levels and fewer observations at the extremes. This pattern aligns with the multinomial logistic regression results, reinforcing ∆TOI as a significant objective marker of spasticity intensity and suggesting potential sex‐based variations in spasticity responses.

By establishing TOI as an objective index, NIRS‐based measurements could offer clinicians a quantitative tool for assessing spasticity severity and monitoring treatment efficacy. Future research should focus on validating these findings in patients with neuromuscular disorders, comparing TOI values with established clinical assessment tools to confirm its clinical utility.

### 4.3 Utility of Ischemia Comparison in Spasticity Measurement

Our results show that the decrease in TOI at 60% contraction intensity (−18.44%) was greater than that observed during induced ischemia (−10.61%). This finding suggests that high‐intensity muscle contractions result in a more pronounced reduction in muscle oxygenation compared to externally applied vascular occlusion. This effect is likely due to a combination of increased metabolic demand and intramuscular pressure restricting local blood flow at higher contraction intensities [17, 21, 22, 23].

It is important to note that both ischemia and 60% MVC contractions were applied for only 30 s in this study. While a general ischemic condition can cause a greater reduction in oxygenation over an extended period, the localized ischemia induced by a 60% MVC contraction occurs more immediately and in a targeted manner. Therefore, within a limited 30‐s timeframe, the more pronounced drop in TOI at 60% MVC compared to ischemia is likely due to the acute restriction of blood flow and increased metabolic demand associated with high‐intensity muscle contractions [20, 23, 24]. Nonetheless, in individuals with spasticity, prolonged and recurrent involuntary contractions may induce even greater metabolic stress over time. As highlighted by Hartkopp et al. [25], severe and frequent lower limb spasms in a paraplegic patient led to significant alterations in muscle fiber composition, with an extreme predominance of slow fibers and near‐complete fatigue resistance—changes reflective of chronic neuromuscular loading. Such findings emphasize that, in some patients, spastic contractions and their associated ischemic burden may approach or even exceed the physiological stress modeled in our study. This highlights the potential of NIRS as a valuable tool for evaluating spasticity‐related ischemia and its effect on muscle metabolism in clinical settings.

### 4.4 Relationship Between TOI and EMG Amplitude

Our data reveal that the correlation between TOI and EMG amplitude weakens as contraction intensity increases. Specifically, at lower intensities (15%–30% MVC), TOI and EMG exhibit a more consistent inverse relationship (correlation coefficient −0.495). However, this relationship becomes less coherent at higher intensities (45%–60% MVC). This decline in correlation may be attributed to several physiological factors, including increased muscle fatigue, altered motor unit recruitment strategies, and localized variations in oxygen utilization within the muscle tissue [26, 27, 28].

During muscle spasms, where contractions occur involuntarily and often with fluctuating intensities, TOI measurements could provide a more stable representation of metabolic demand compared to EMG, which primarily captures electrical activity without direct metabolic correlation. Given that our results show that TOI reductions at high contraction levels exceed those seen in ischemia, it is plausible that TOI could serve as a more robust marker of muscle spasticity severity than EMG alone.

While EMG effectively detects electrical activity in active muscles, it fails to capture muscle state changes when the muscle is quiescent [27, 28]. Figure 2 demonstrates that even when EMG signals are absent, NIRS continues to detect fluctuations in muscle oxygenation. This suggests that NIRS could track the full cycle of a spasm—from initiation to resolution—offering a continuous, objective measure of muscle spasticity. Integrating NIRS with EMG could enhance diagnostic precision by providing both electrical and hemodynamic data, allowing for a more comprehensive analysis of muscle spasms.

### 4.5 Limitations and Future Directions

Despite the promising results, our study has limitations. The study was conducted on healthy individuals aged 21–57. Future research should include more diverse populations to explore potential differences between different age ranges (children, adults, seniors). Furthermore, future studies should include patients with various neuromuscular conditions to validate the generalizability of the findings.

Future studies should explore potential applications of NIRS in clinical scenarios that affect muscle hemodynamics, such as in the assessment of muscle spasticity in various neuromuscular conditions, such as children with CP and stroke patients. Additionally, research should also investigate the long‐term use of NIRS in monitoring chronic muscle conditions and its potential application in guiding therapeutic interventions.

## 5 Conclusion

Our findings highlight the potential of NIRS as an objective, non‐invasive tool for assessing muscle spasticity. By quantifying muscle oxygenation changes in real time, NIRS could provide clinicians with a reliable alternative to subjective spasticity assessment scales. The demonstration of TOI as a predictive index for spasticity assessment, supported by logistic regression modeling, strengthens the case for its clinical application. Further studies are required to validate these findings in muscle spasticity‐affected populations and to optimize the technology for real‐world clinical use.

## Conflicts of Interest

The authors declare no conflicts of interest.

## Data Availability

The data that support the findings of this study are available from the corresponding author upon reasonable request.
